# *Accacoelium contortum* (Trematoda: Accacoeliidae) a trematode living as a monogenean: morphological and pathological implications

**DOI:** 10.1186/s13071-015-1162-1

**Published:** 2015-10-15

**Authors:** Ana Elena Ahuir-Baraja, Francesc Padrós, Jose Francisco Palacios-Abella, Juan Antonio Raga, Francisco Esteban Montero

**Affiliations:** Cavanilles Institute of Biodiversity and Evolutionary Biology, Marine Zoology, Science Park, University of Valencia, Catedrático José Beltrán, 2, 46980 Paterna, Valencia Spain; Fish Diseases Diagnostic Service. Departament de Biologia Animal, de Biologia Vegetal i d’Ecologia, Facultat de Veterinaria, Universitat Autònoma de Barcelona, 08193 Barcelona, Cerdanyola Spain

**Keywords:** *Mola mola*, Accacoeliid, Ectoparasitism, Morphology, Immature specimens, Pathology, Adaptation

## Abstract

**Background:**

*Accacoelium contortum* (Rudolphi, 1819) Monticelli, 1893 is a frequent but poorly known trematode found on gills, pharynx and digestive tract of the ocean sunfish *Mola mola* (L.). Although the morphology of *A. contortum* agrees with that of a typical endoparasitic trematode, with two relatively small suckers and no large holdfasts, this parasite is normally ectoparasitic. The main objective of this paper is to explore this peculiar host-parasite relationship.

**Methods:**

A total of 106 ocean sunfish were examined for the presence of *A. contortum*. The oropharyngeal chamber (gills and pharynx) and the digestive tract were analysed. As the previous descriptions of this species seem to be based on contracted specimens, for the morphological study the parasites were killed using two methods: with hot 70 % ethanol (with relaxed bodies) and with 70 % ethanol at room temperature (with contracted bodies). For histological studies, samples from fresh fish with parasitised left gills, pharynx and digestive tract were fixed in buffered 10 % formalin. For molecular studies the 18S, 28S and ITS-2 sequences were provided and compared with the available data in GenBank®.

**Results:**

New information on the morphology of *A. contortum* and on the parasite-related response and pathological alterations in the host are given. New diagnostic traits for some structures are provided: e.g. tegumental papillae of the forebody with apical digitiform swellings and mouth surrounded by a circum-oral crown of simple papillae. The length of the ventral sucker peduncle and the position of the vitellarium were found to be associated with the contraction degree of the specimen. Immature individuals of this species are described for the first time. An intense proliferative inflammatory response of host gill and pharynx epithelium at the host-parasite interface was detected and parasites became partially covered by overgrowths of host tissues.

**Conclusions:**

The induction of prominent histological alterations associated with *A. contortum* seems to be an adaptation to the external environment, an unusual location for trematodes.

## Background

The ocean sunfish, *Mola mola* (L.) (Tetraodontiformes, Molidae), is the largest bony fish of the world [1, 2]. Its unique size and morphology has increased the interest of large public aquariums in keeping this species in their facilities. However, husbandry of ocean sunfish is very complicated due to the fact that this species is very sensitive to stress [3], together with the high prevalence and abundance of parasites on fish [4–8]. Surprisingly, information about the effect of the parasites on the ocean sunfish is very scarce. One of the most frequent parasites is the trematode *Accacoelium contortum* (Rudolphi, 1819) Monticelli (Accacoeliidae Odhner, 1911) a poorly known parasite mainly located within the oropharyngeal chamber (on gills, oral cavity and pharynx) provoking important alterations of the host tissues. The Accacoeliidae includes parasitic hemiuroids mostly reported in *M. mola* and *M. ramsayi* (Giglioli, 1883) [9–11]. *Accacoelium contortum* is the type-species of the family and the only known species of the genus *Accacoelium* Monticelli, 1893. This trematode has been reported in ocean sunfish from the Mediterranean, North East Atlantic and South Pacific [4, 9–11], with a single record in blotched picarel, *Spicara maena* (L.) (Centracanthidae) [12]*. Accacoelium contortum* is mostly an ectoparasite, although it has also been reported within the oesophagus and digestive tract [4, 6, 8, 9, 11, 13–17]. The general external morphology and attachment organs of *A. contortum* are very similar to those of the rest of the species of the family (and that of most trematodes); elongated body with oral and ventral suckers and papillate forebody. In this regard, the ectoparasitic behaviour of this species is an exception among the Accacoeliidae, and very infrequent within the class Trematoda (mostly endoparasites) [9]. Despite being unique in its kind, little is known about the biology of *A. contortum*, especially its attachment strategy.

During an extensive parasitological survey on *M. mola* from the Western Mediterranean, specimens of *A. contortum* were detected with high prevalence and intensity and this was usually associated with severe alterations of fish tissues. This provided us with the opportunity to obtain detailed morphological data for *A. contortum*, report new morphological traits and describe immature individuals of this species for the first time. In the present work we also describe the biology of this trematode and the special relationship between the host and the parasite. In the same way, the pathological effects of the parasites on its host are described, also discussing how the parasite adapts to the external environment.

## Methods

In total, 106 ocean sunfish [total length × total height (mean ± standard deviation with range in parentheses): 44.0 ± 11.7 (33.0 − 107.0) × 64.5 ± 11.9 (58.0 − 140.0) cm] were captured as by-catch in the ‘*almadraba*’ (a traditional netting technique dedicated to the capture of tunas) off La Azohía, Cartagena (Spain) (1°15’ W, 37°33’ N) during May and June, from 2005 to 2008. All fish were frozen at −18 °C, except for 30 fish that were analysed fresh 3–4 h after capture. Fresh fish were analysed at the laboratory of the Spanish Institute of Oceanography in Puerto de Mazarrón (Cartagena, Spain), while thawed fish were analysed in the facilities of the Marine Zoology (MZ) of the Cavanilles Institute of Biodiversity and Evolutionary Biology, University of Valencia (Valencia, Spain). Parasites on mouth, gills, pharynx and digestive tract were collected and identified in saline solution under stereomicroscope Leica MZ AP0 (up to 80×). Total abundance (TA), prevalence (P in %) and mean intensity (±standard deviation) (MI) were calculated according to Bush et al. [18]. Confidence intervals of prevalence (CI) were calculated using Quantitative Parasitology 3.0 [19]. Parasite abundance in the right and left gills was compared with Wilcoxon test using SPSS V. 15.0. (SPSS, Inc., London, UK) [20].

For morphological descriptions, some of the parasites collected from fresh fish were fixed and mounted to be examined under light microscopy and scanning electron microscopy (SEM). It is widely recognised that the descriptions of helminths must not be based on contracted specimens [21]; however, previous descriptions of *Accacoelium contortum* seem to be based on contracted specimens. In order to compare contracted and relaxed specimens morphologically, parasites were killed and fixed at different temperatures, resulting in different degrees of contraction [22]. Therefore, specimens were classified according to the method of killing as either “relaxed” specimens (killed in hot 70 % ethanol, with relaxed bodies) or as “contracted” specimens (killed in 70 % ethanol at room temperature, with contracted bodies). Twenty specimens of each type (“relaxed” and “contracted”) were stained with iron acetocarmine (for 15–20 min), rinsed in 1 % HCl in 70 % ethanol, dehydrated in an ethanol series (70 to 100 %), transferred to 98 % dimethyl phthalate as clearing agent, and mounted in Canada balsam in lateral view. Morphological description is based on relaxed specimens only. Sucker dimensions (length, width, and depth) were taken from unmounted specimens, as the specimens were mounted laterally because of the protuberant ventral sucker, preventing measurement of the width of the acetabulum. In order to confirm our observations, we also examined vouchers deposited in the British Museum (Natural History) Collection at the Natural History Museum (BMNH) (London, UK): two stained and mounted specimens and histological sections of *A. contortum* (Acc. Nos. 1973.1.25.17 − 20). New voucher specimens from the present study were deposited in the BMNH collection (2 vouchers NHMUK. 2015.9.24.1 − 2 *A. contortum*) and in the MZ collection (3 vouchers UV/ZOOMAR/*M. mola*/ 12034–12035 (with two specimens in one of the slides) *A. contortum*).

Drawings were made with the aid of a drawing tube attached to a light microscope Nikon Optiphot − 2 (Nikon Corporation) up to 1000 × magnification. Measurements were taken using Image Tool 3.00 (developed at the University of Texas Health Science Centre at San Antonio and available at http://ddsdx.uthscsa.edu/dig/itdesc.html) and are given in millimeters (with the mean ± SD in parentheses) unless otherwise stated.

For SEM studies, two specimens were fixed in 10 % buffered formalin, dehydrated in an ethanol series, critical point dried in liquid CO_2_, mounted on specimen stubs with conductive carbon paint, sputter coated with gold-palladium to a thickness of 25–30 nm in a Bio Rad–Sc 500 coating unit, and examined in a HITACHI S–4100 scanning electron microscope at 5 kV.

For histological studies, samples from fresh fish with parasitised and non-parasitised left gills, pharynx and digestive tract were fixed in buffered 10 % formalin. Gills and pharynx were previously decalcified in 8 % formic acid for 72 h, replacing the formic acid every 24 h. All samples were placed in cassettes, dehydrated and embedded in paraffin, serially sectioned at 4 μm with a rotary microtome, stained with haematoxylin and eosin (H-E), mounted in Entellan™(Merck) and observed under light microscopy Leica DMR HC microscope (magnifications up to 400×). Some sections were stained with Giemsa to detect the presence of secondary bacterial infections.

For the molecular analysis the total DNA was isolated from single adults (seven specimens contracted and relaxed from gills and pharynx) using the DNeasy® Blood & tissue Kit (Quiagen, Venlo, The Netherlands). Three DNA genes were amplified via polymerase chain reaction (PCR): the 18S rDNA, the 28S rDNA and the internal transcribed spacer (ITS-2) rDNA. The 18S was amplified in three fragments with the primer pairs: Worm A (forward; 5´-GCG AAT GGC TCA TTA AAT CAG-3´) and A27 (reverse; 5´-CCA TAC AAA TGC CCC CGT CTG-3´); 600 F (forward; 5´-GGT GCC AGC MCG GGC G-3´) and 1420R (reverse; 5´-TAA CCA GAC AAA TCG CTC C-3´); 930 F (forward; 5´-GCA TGG AAT AAT GGA ATA GG-3´) and Worm B (reverse; 5´-CTT GTT ACG ACT TTT ACT TCC-3´) [23, 24]. The 28S was amplified in two fragments with the primer pairs: U178 (forward, 5´-GCA CCC GCT GAA YTT AAG-3´) and ECD2 (reverse, 5´-CCT TGG TCC GTG TTT CAA GAC GGG-3´); 300 F (forward, 5´-CAA GTA CCG TGA GGG AAA GTT G-3´) and 1200R (reverse, 5´-GCA TAG TTC ACC ATC TTT CGG-3´) [25, 26]. And the ITS-2 was amplified with the primer pairs: GA1 (forward; 5´-AGA ACA TCG ACA TCT TGA AC-3´) and ITS2.2 (reverse; 5´-CCT GGT TAG TTT CTT TTC CTC CGC-3´) [27]. The thermocycling profile of the PCR amplifications consisted of an initial DNA denaturation at 95 °C for 2 min, followed by 35 cycles of amplification at 95 °C for 50s, annealing at 55 °C for 50 s and extension at 72 °C for 2 min, and final extension at 72 °C for 7 min, with the exception of amplifications for 28S which were carried out with an annealing temperature of 52 °C (modified from [28]).

The amplicons were purified with the NucleoSpin Gel and PCR Clean-up kit (Machery-Nagel, Düren, Germany), following the manufacturer’s instructions and sequenced by Macrogen Europe Inc. (Amsterdam, The Netherlands) on a 3730XL DNA analyzer (Applied Biosystems, Foster City, USA) using the primers used for PCR. Consensus sequences were assembled using Bioedit 7.2.3 [29] and a representative sequence for each gene was submitted to BLAST on GenBank® database under the accession numbers KT005302 for 18S, KT005303 for 28S and KT005304 for ITS-2.

## Results

**Fig. 1:**
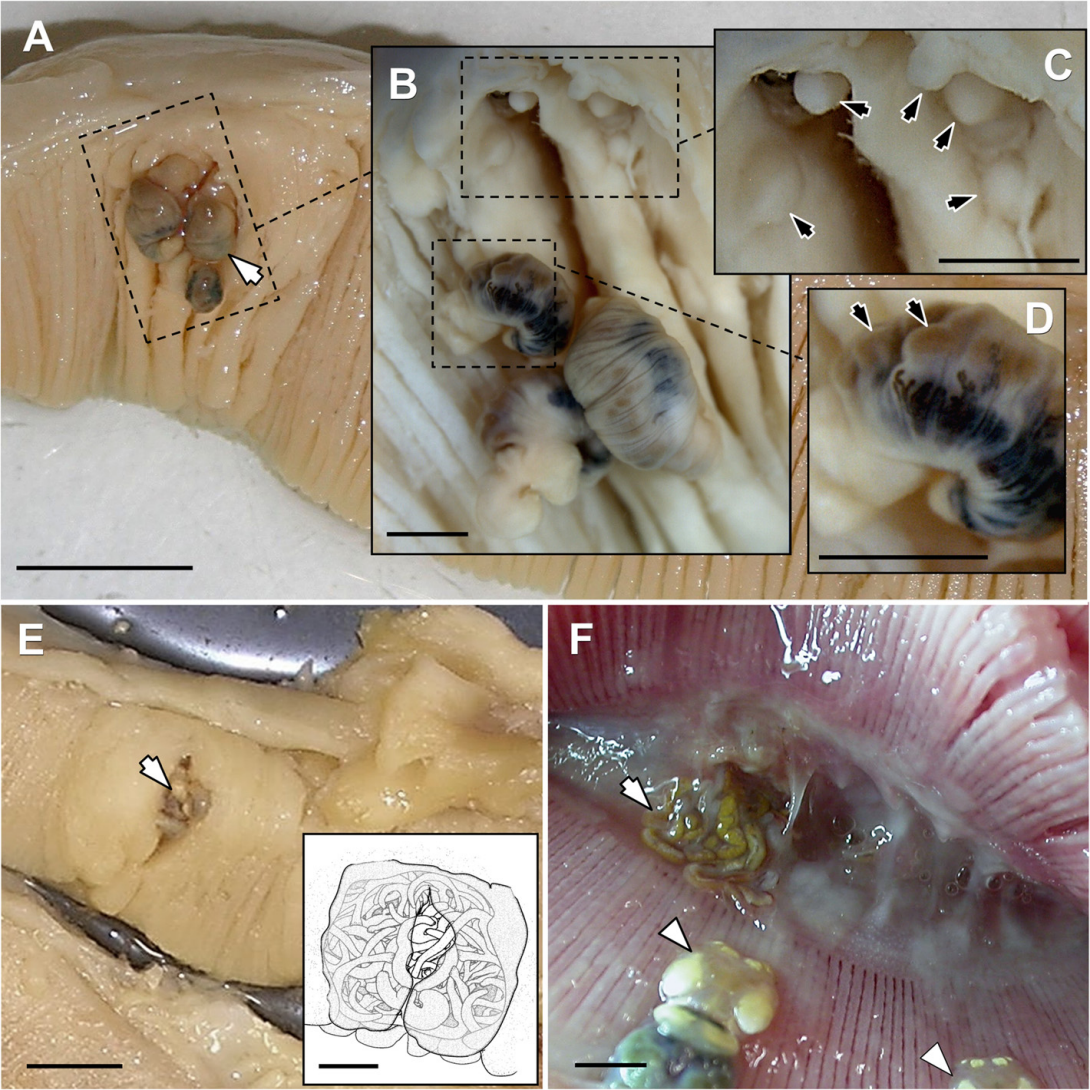
Infection sites of *Accacoelium contortum* in the ocean sunfish *Mola mola* (L.) from the Western Mediterranean. **a** Gill infected with *A. contortum* with parasites attached (arrow); scale bar = 1 cm. **b** Detail of (**a**) showing the infection area with parasites detached, scale bar = 2 mm. **c.** Detail of (**b**) showing little swellings (arrows) on gill tissue caused by *A. contortum* sucker vacuum; scale bar = 2 mm. **d** Detail of (**b**) showing little swellings (arrows) in the parasite surface caused by *A. contortum* suckers; scale bar = 2 mm. **e** Gill infected with numerous *A. contortum* clustered inside a pit (arrow) within swollen tissue (inset: a schematic representation where gill tissues covering the parasites are portrayed as semi-transparent in order to show the cluster of parasites below); scale bars = 1 cm (inset 2 mm). **f** Specimens of *A. contortum* on the pharynx; note that the cluster of parasites is disaggregated and several parasites lie over the pharyngeal teeth and gill arch (arrow). The gills were also infected by the copepod *Cecrops latreillii* (arrowheads); scale bar = 2 cm

Nearly half of the ocean sunfish examined (*P* = 47.2 %, CI: 37.7 − 56.7 %) were parasitised by *A. contortum* (TA = 556; MI = 10.8 ± 16.8). Parasites were mainly found on gills (*P* = 35.8 %, CI: 27.2 − 45.7 %; TA = 265; MI = 7.2 ± 8.0) and pharynx, among the pharyngeal teeth (*P* = 17.9 %, CI: 11.7 − 26.3 %; TA = 262; MI = 13.8 ± 25.2) (see Fig. 1). Furthermore, a few specimens were found in the stomach (*P* = 7.5 %, CI: 3.5 − 14.4 %; TA = 29; MI = 3.3 ± 2.8) (four fish had only *A. contortum* in this location). Four immature specimens were also found in the stomach of one fish. A significant difference in the abundance of *A. contortum* between the right and left gills was observed (Wilcoxon, Z = −4.8; *p* < 0.05), with higher abundance in the right gills.

### Diagnostic traits

The morphology of the newly collected parasite specimens from the Western Mediterranean ocean sunfish is in accordance with previous descriptions of *A. contortum*, as well as with the museum vouchers. However, our examination of the newly collected specimens enabled us to add further morphological detail in order to help to identify the species. A description of the immature specimens, unreported up to date, is included.

#### Description of mature adults of A. contortum (Fig. 2a–g)

**Fig. 2:**
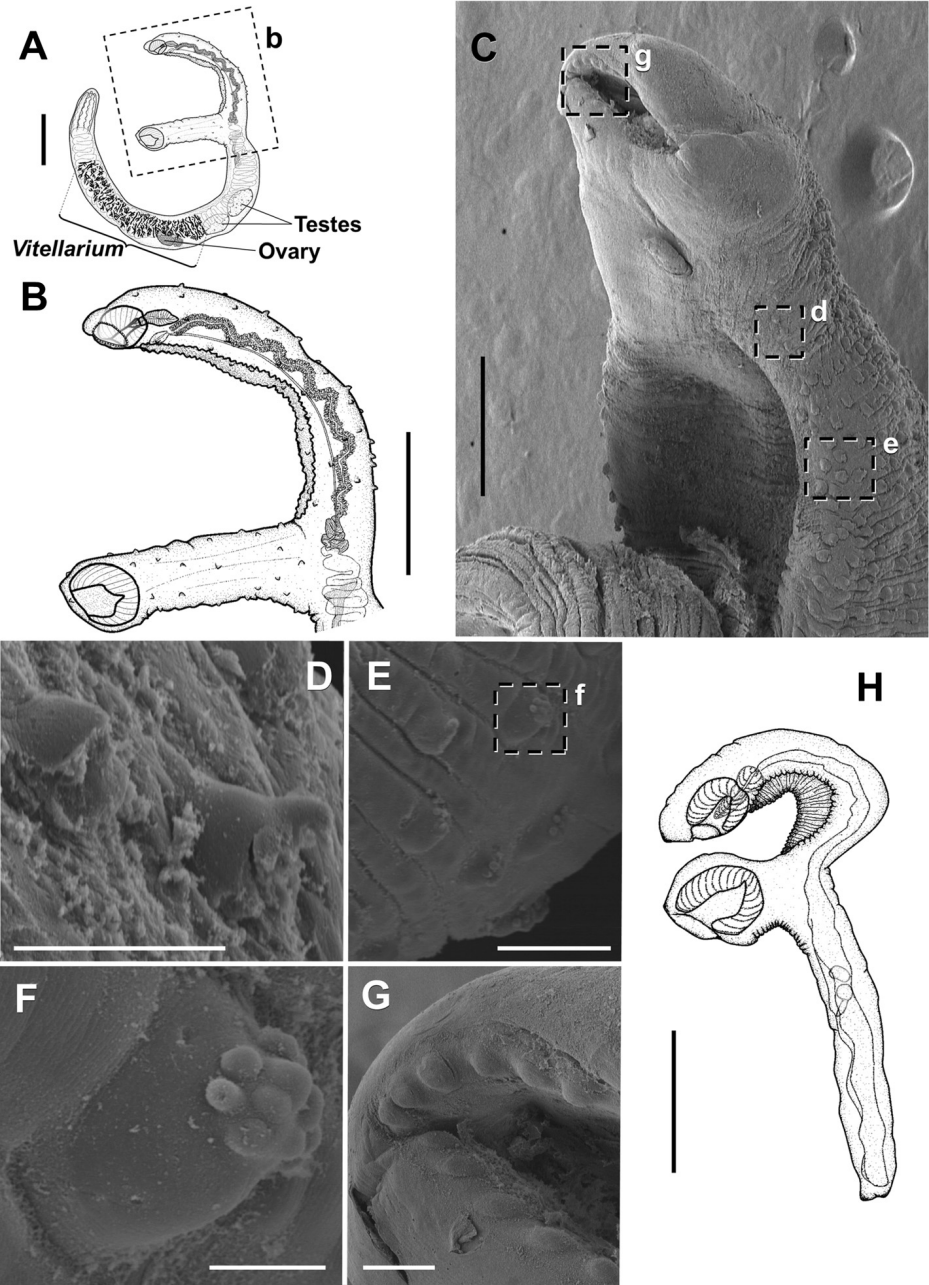
Specimens of *Accacoelium contortum* in the ocean sunfish *Mola mola* (L.) from the Western Mediterranean. **a** Adult relaxed specimen in lateral view (*b* indicates the area magnified in Fig. 2b); scale bar = 1 mm. **b** Detail of the anterior extremity showing the long peduncle of the ventral sucker; scale bar = 1 mm. **c** Detail of the ventro-lateral view of the forebody (SEM) (*d, e* and *g* indicate the papillae magnified in (**d**), (**e**) and (**g**), respectively); scale bar = 600 μm. **d** Tegumental papillae of the forebody with a single swelling at the tip; scale bar = 50 μm. **e** Group of tegumentary papillae of the forebody with several apical swellings (*f* indicates the papilla magnified in (**f**); scale bar = 100 μm. **f** Detail of a tegumental papilla with a cluster of digitiform swellings at the tip; scale bar = 20 μm. **g** Detail of the crown of circum-oral papillae; scale bar = 70 μm. **h** Immature specimen in lateral view; scale bar = 500 μm

General morphology as in Bray and Gibson [9]; additional morphological comments follow. Forebody with a ventral depression with transverse folds, extending from oral sucker to base ventral sucker peduncle (Fig. 2a-c). Tegument of forebody (including ventral sucker peduncle) bearing conical to dome-shaped papillae [30.4 − 49.2 (38.0 ± 7.5) μm wide at the base, *n* = 20] (Fig. 2c-f), with numerous distal short digitiform swellings at the tip (apparently less numerous anteriorly, where sometimes only single swelling observed at the tip, see Fig. 2d). A single crown of circum-oral papillae present (Fig. 2g); papillae single, conspicuous, dome-shaped [25.1 − 33.4 (30.9 ± 2.9) μm wide at base (*n* = 20)], 34–36 in number (counted in SEM micrographs). Oral sucker ventro-subterminal, with soft pre-oral lobe (Fig. 2a, b). Ventral sucker on a long peduncle in relaxed specimens (0.9 − 3.8 (2.2 ± 0.9) long (not including sucker) (Fig. 2a, b); ventral sucker peduncle to body length ratio of 1:4.0 − 20.6 (*n* = 20). Peduncle very short in contracted specimens (0.1 − 0.4 (0.2 ± 0.1) long (not including the sucker) (see Figure two in Gibson and Bray [9]), with ventral sucker peduncle to body length ratio of 1:24.1 − 76.0 (*n* = 20) (see Table 1 for morphometric data of relaxed and contracted specimens). Pars prostatica moderately sinuous in relaxed specimens and strongly coiled in contracted specimens (see Figure two in Gibson & Bray [9]). Position of anterior extremity of vitellarium with respect to testes variable (*n* = 24): anterior extremity of vitellarium posterior to posterior testis in 11 specimens, anterior to posterior testis in 5, at mid-length of posterior testis in 4, at mid-length of anterior testis in 2, and anterior to anterior testis in 2. Posterior extremity of vitellarium always extending beyond the ovary, up to posterior extremity of body.

**Table 1:** Morphometric data for relaxed and contracted specimens of *Accacoelium contortum* from the ocean sunfish *Mola mola* (L.) examined in the present study

Morphometric features	Relaxed specimens (*n* = 20)	Contracted specimens (*n* = 20)
Body length	8.9–20.5 (15.6 ± 3.1)	5.5 − 15.6 (10.0 ± 3.0)
Maximum body width	0.6– 1.9 (1.2 ± 0.3)	0.7–2.1 (1.2 ± 0.3)
Oral sucker^a^	0.4–0.6 (0.5 ± 0.1) × 0.4–0.5 (0.5 ± 0.2) × 0.4–0.6 (0.5 ± 0.1)	0.5 − 0.6 (0.5 ± 0.1) × 0.5–0.6 (0.5 ± 0.1) × 0.4–0.7 (0.5 ± 0.1)
Ventral sucker^a^	0.6–0.8 (0.7 ± 0.1) × 0.4–0.7 (0.6 ± 0.2) × 0.7–1.1 (0.8 ± 0.1)	0.4–0.7 (0.6 ± 0.1) × 0.5–0.6 (0.5 ± 0.2) × 0.7(1.2 (0.8(0.2)
Ventral sucker peduncle length	0.9–3.8 (2.2 ± 0.9)	0.1–0.4 (0.2 ± 0.1)
Ventral sucker peduncle width	0.6–1.2 (0.7 ± 0.2)	0.8–1.3 (1.1 ± 0.2)
Pharynx^b^	0.5–0.8 (0.6 ± 0.1) × 0.2–0.3 (0.2 ± 0.0)	0.4–0.5 (0.5 ± 0.0) × 0.1–0.3 (0.2 ± 0.0)
Maximum testis diameter	Anterior testis: 0.7–1.5 (1.1 ± 0.2)	Anterior testis: 0.5–0.8 (0.7 ± 0.1)
	Posterior testis: 0.8–1.4 (1.1 ± 0.2)	Posterior testis: 0.6–0.9 (0.8 ± 0.1)
Minimum testis diameter	Anterior testis: 0.4–1.1 (0.8 ± 0.2)	Anterior testis: 0.3–0.5 (0.5 ± 0.1)
	Posterior testis: 0.4–1.1 (0.8 ± 0.2)	Posterior testis: 0.3–0.7 (0.5 ± 0.2)
Maximum ovary diameter	0.4–1.0 (0.7 ± 0.2)	0.3–0.6 (0.4 ± 0.1)
Minimum ovary diameter	0.3–0.7 (0.5 ± 0.1)	0.2–0.6 (0.4 ± 0.1)
Eggs^b^	0.02–0.03 (0.03 ± 0.0) × 0.01–0.02 (0.02 ± 0.0)	0.02–0.03 (0.03 ± 0.0) × 0.01–0.02 (0.02 ± 0.0)

All measurements are in millimetres

^a^length × width × depth

^b^length × width

**Fig. 3:**
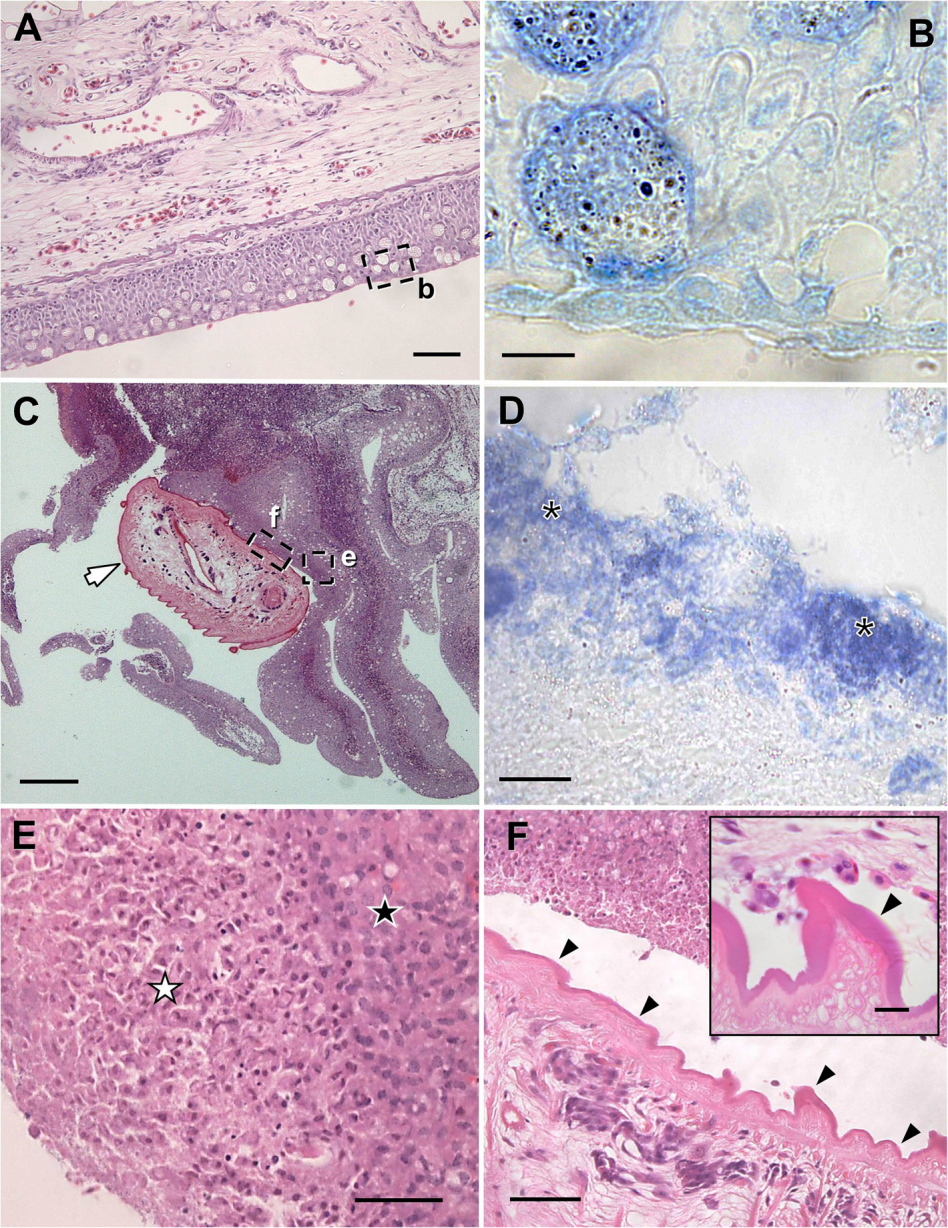
Histology of infections by *Accacoelium contortum* in gills of the ocean sunfish *Mola mola* (L.) from the Western Mediterranean. **a** Uninfected gills (*b* refers to area magnified in (**b**); scale bar = 100 μm. **b** Gill epithelium without secondary bacterial infections (stained with Giemsa); scale bar = 10 μm. **c** Gills showing the parasite (arrow) surrounded by large papillary folds (*e* and *f* indicate the areas of infection magnified in **e** and **f**, respectively); scale bar = 200 μm. **d** Secondary bacterial infections (asterisks) in the gill epithelium associated with *A. contortum* infections (stained with Giemsa); scale bar = 10 μm. **e** Detail of Fig. 3c showing the area of the gill tissue in contact with the parasite surface with thick pale-pink layer (white star) and pyknotic necrotic epithelial cells (black star); scale bar = 50 μm. **f** Detail of (**c**) showing the outer dense homogeneus layer covering parasite tegument (arrows) (inset: enlarged papilla showing a detail of this layer); scale bar = 50 μm (inset 10 μm). Stained in H-E, except for (**b** and **d**)

In histological sections, the nucleate and anucleate external layers of the tegument are covered with a thick outer dense homogenous layer, particularly thicker over the parasite papillae (see detail in Fig. 3f).

#### Description of immature A. contortum

Body elongate 1.6 − 1.9 (1.8 ± 0.1) long, 0.1 − 0.2 (0.1 ± 0.01) wide (*n* = 4) Fig. 2h). Tegumental papillae not observed. Forebody with strongly folded ventral depression. Oral sucker ventro-subterminal, 0.1 (0.1 ± 0.0) long, 0.1 (0.1 ± 0.06) wide, 0.1 − 0.2 (0.2 ± 0.02) deep. Pre-oral lobe present. Ventral sucker, 0.2 − 0.3 (0.2 ± 0.04) long, 0.2 (0.2 ± 0.02) wide, 0.2 − 0.3 (0.2 ± 0.05) deep, on a peduncle (length of peduncle depends on the degree of contraction of the specimens), 0.05 − 0.12 (0.09 ± 0.04) long, 0.16 − 0.23 (0.20 ± 0.04) wide at mid-length. Pharynx elongate-piriform, 0.14 − 0.16 (0.15 ± 0.01) long, 0.04 − 0.06 (0.05 ± 0.01) wide, extending into oral sucker. Genital primordium observed in some specimens.

The newly-generated sequences were aligned and compared with sequences of 18S rDNA and the partial 28S rDNA for *A. contortum* from GenBank® (AJ287472 and AY222190 respectively) [23, 28, 30]. Both 18S (1698 pb) and 28S (924 pb) sequences for isolates of *A. contortum* were identical to the GenBank® sequences. The ITS-2 (480pb) sequence was also submitted for the first time to GenBank® (accession numbers: KT005302 for 18S, KT005303 for 28S and KT005304 for ITS-2).

### Pathology related to *Accacoelium contortum*

Parasites located on gills and pharynx were always clustered in groups (Fig. 1), more numerous in the pharynx: 13–36 parasites per cluster on gills (Fig. 1a, e) and 29–93 parasites per cluster on pharynx (Fig. 1f). Suckers attached them to the host tissues and to other parasites, while the hindbodies of several parasites were rolled around other individuals. When parasites were removed, the round swellings caused by the vacuum action of the suckers were visible on fish tissues and on parasite surfaces (Fig. 1b-d). In the areas surrounding parasite clusters, different types of macroscopic alterations such as tissue proliferations, swellings or malformations were observed (Fig. 1a, e).

On the gills, parasites were often located in pits between deformed and bent gill filaments and surrounded by gill tissue proliferations (Fig. 1e).

In the histological sections, the surrounding areas where *A. contortum* specimens were attached were observed to display extensive regions with intense proliferative and inflammatory response (Fig. 3a and c show uninfected and infected tissues, respectively). Parasites were surrounded by large papillary folds associated with intense epithelial hyperplasia and infiltration with inflammatory cells of the subepithelial connective tissue (Fig. 3e). Epithelial hyperplasia was characterised by an intense proliferation of the epithelial layers and also by mucous cells, mainly in the papillary folds. In the areas of the host-parasite interface, an external thick pale-pink layer and pyknotic necrotic epithelial cells were observed (Fig. 3e), and epithelium was often highly eroded or even absent. Clusters of bacterial colonies were revealed, in preparations stained with Giemsa, in the external layer closely associated to the necrotic debris surrounding the infected tissues (Fig. 3b and d show uninfected and infected tissues, respectively).

**Fig. 4:**
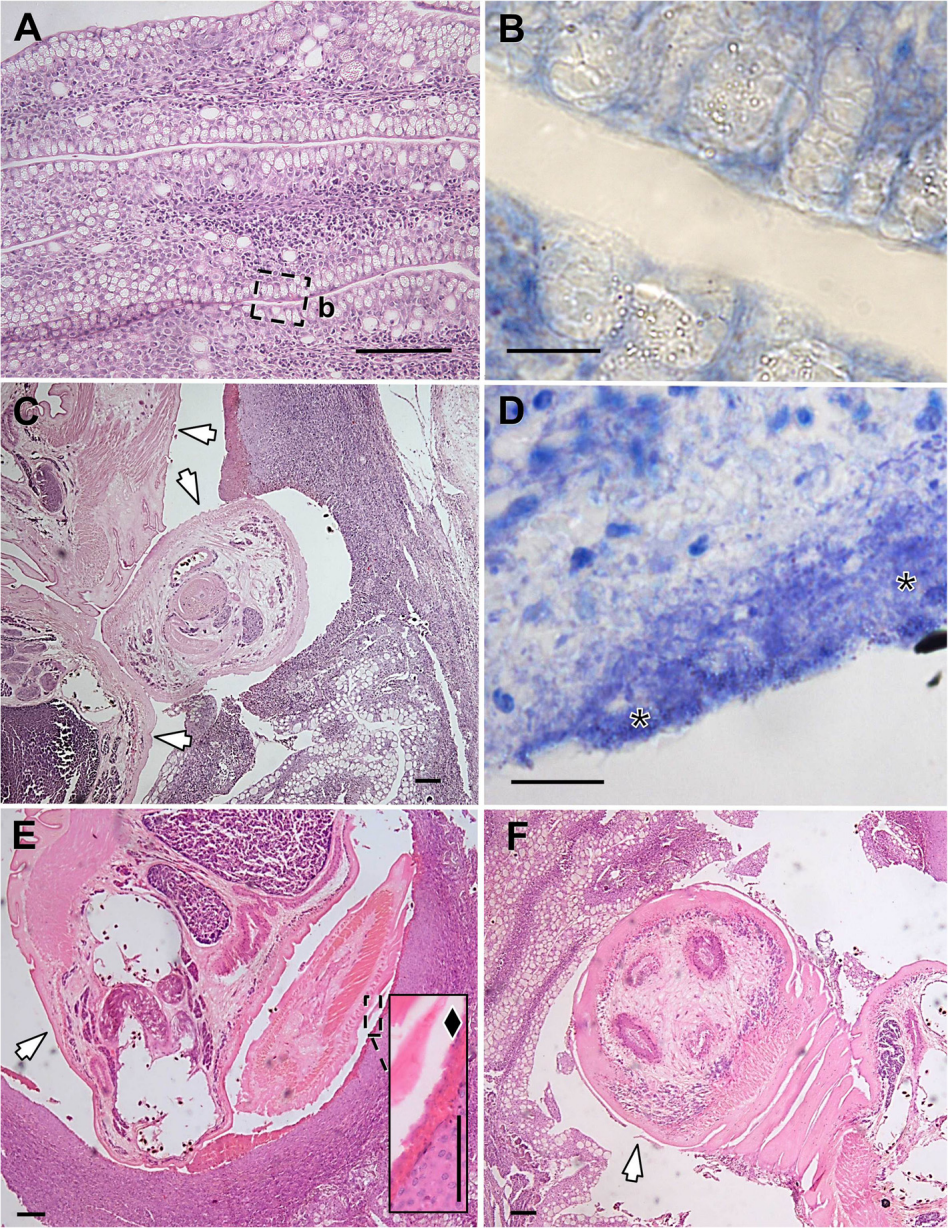
Histology of infections by *Accacoelium contortum* in pharynx of the ocean sunfish *Mola mola* (L.) from the Western Mediterranean. **a** Uninfected pharynx (*b* refers to area magnified in (**b**); scale bar = 100 μm. **b** Pharyngeal epithelium without secondary bacterial infections (stained with Giemsa); scale bar = 10 μm. **c** Infected pharynx showing three parasites (arrows), one of them partially covered by the pharyngeal epithelium; scale bar = 100 μm. **d** Secondary bacterial infections (asterisks) in the pharyngeal epithelium associated with *A. contortum* infections (stained with Giemsa); scale bar = 10 μm. **e.** *A. contortum* with a specific eosinophilic compact outer layer in the epithelium in contact with the parasite forebody (arrow) (inset: detail of the layer (black diamond)); scale bar = 100 μm (inset 50 μm). **f** *A. contortum* hindbody (arrow) in contact with the pharyngeal epithelium; scale bar = 100 μm. Stained in H-E, except for (**b** and **d**)

In the pharynx, parasites were also enclosed in pits within large tissue proliferations displaying similar characteristics as described before (Fig. 4a and c show uninfected and infected tissues, respectively). Affected pharyngeal epithelium also displayed intense hyperplasia and sometimes necrotic external layers. In some cases, pharyngeal epithelium displayed a specific eosinophilic compact outer layer in contact with the parasite forebody (see detail in Fig. 4e), where the tegumental papillae are located (see additional diagnostic traits for the parasite). The compact outer layer was not observed on pharyngeal epithelium in contact with the parasite hindbody (Fig. 4f). Preparations stained with Giemsa revealed a secondary bacterial infection (Fig. 4b and d show uninfected and infected tissues, respectively).

The parasites located in the stomach were always scattered, and were neither clustered nor related to apparent changes in the nearby epithelium.

## Discussion

Most of the mature specimens of *Accacoelium contortum* occurred in the oropharyngeal chamber (gills, oral cavity and pharynx), and only a few disperse mature and immature parasites were found within the stomach. Interestingly, parasite abundance was significantly higher in the right gills than in the left gills. This asymmetry is not common in gill parasites, and only some authors have reported differences in parasite abundance between left and right gills, e.g. in monogeneans [31, 32] or copepods [33]. It is very difficult to find anatomical reasons to explain this because fish are bilaterally symmetrical. A possible explanation in ocean sunfish could be related to fish behaviour, as this species tends to incline the body laterally when resting on the surface [34–36] or when swimming in the water column [34, 36–39]. These studies indicated that ocean sunfish tend to incline the bodies mainly to the right when they are ascending, implying that they mostly expose the left side to the air when they have arrived at the surface to rest. Parasites in the left side would be in these moments submitted to partial desiccation, heating or UV exposure that could somehow compromise their survival.

The molecular analysis confirms that all morphotypes, relaxed and contracted, belong to the same species, *A. contortum*. This study includes several new diagnostic morphological features. The different morphology of the tegumental papillae on the forebody is described in detail and the circum-oral papillae are described for the first time. Moreover, the peduncle of the ventral sucker of *A. contortum* seems to be of variable length and the latter cannot be described as “short penduncled” or “sessile” as was indicated previously [9, 10]. However, the absolute and relative values of the length of the ventral sucker peduncle in the relaxed specimens are the highest values among the accacoeliids reported to date: up to 3.8 mm in length and a peduncle to body length ratio of up to 1:4; this is in contrast with the data from contracted specimens with very short penducles of 0.1 − 0.4 mm in length and a peduncle to body length ratio of 1:24–76 (including measurements from the BMNH vouchers). *Rhynchopharynx paradoxa* Odhner, 1928 is the only accacoeliid with a markedly developed peduncle of the ventral sucker marked: up to 3 mm in length and with a peduncle to body length ratio of 1:9 in apparently relaxed specimens (see Figure nine in Bray & Gibson [9]) [10, 40]. According to our observations, the ventral sucker peduncle of *A. contortum* is extensible and remains elongated or contracted depending on the degree of relaxation or contraction of the specimens*.* An extensible ventral sucker peduncle has been described for the species of another accacoeliid genus, *Accacladium* Odhner, 1928. Regarding the arrangement of the vitellarium, according to Bray & Gibson [9], Gibson & Bray [10] and Gibson [40], one of the diagnostic traits of the genus *Accacoelium* is that the vitellarium is posterior to the anterior testis. However, some specimens examined here possessed a vitellarium located anteriorly to the anterior testis. The arrangement of this organ seemed to be related with the degree of contraction of the specimens. Nonetheless, *A. contortum* still appears to be the only species of the Accacoeliinae Odhner, 1911, with vitellarium extending profusely posterior to the ovary. This trait is therefore a key diagnostic trait for the genus *Accacoelium*. The newly corrected morphological traits must be taken into account to avoid confusion when differentiating this genus and species from among the other accacoeliids. Accordingly, a modified description of *A. contortum* must include the following diagnostic traits: single circum-oral crown of tegumental papillae present; ventral sucker on a long peduncle in relaxed specimens; anterior extremity of vitellarium from anterior to anterior testis to immediately posterior to posterior testis, posterior extremity always extending beyond the ovary.

We also provide the first description of immature individuals of *A. contortum*. The morphology of immature individuals was similar to adult specimens, except for the presence of primordial genitalia and the absence of tegumental papillae on the forebody and the circum-oral rounded papillae. Immature individuals were only found in the stomach of one fish, where parasites would have been recruited by trophic transmission. Although the metacercariae of *A. contortum* have not been described yet, those of other accacoeliids have been recorded in planktonic animals [9], the main food for ocean sunfish (mostly gelatinous zooplankton as coelenterates, ctenophores and salps [36, 41]). The stomach contents of the sunfish herein studied were analysed in order to find metacercariae, but no parasites were found, probably due to the fast digestion of this kind of prey.

Parasites on gills and mouth are conventionally considered as ectoparasites, as they are surrounded by water, exposed to external conditions. As previously stated, the external microhabitat of *A. contortum* is exceptional among the trematodes and unique among the accacoeliids. Perhaps, the most well-known example of ectoparasitic trematodes of fish are the transversotrematids, a family with several species, all of them adapted to live under fish scales [42, 43]. Apart from *A. contortum*, other parasites of the superfamily Hemiuroidea Looss, 1899, such as some syncoeliids, have been occasionally found in the skin and buccal and branchial cavities of marine teleosts and elasmobranchs, although these sites are often considered as doubtful permanent locations [40, 44]. Due to their inherited bauplan, *A. contortum* displays the morphology of an endoparasitic trematode, with a pair of simple suckers, unsuitable for open microhabitats and water streams such as those in the oropharyngeal chamber. Other attachment strategies seem to have been developed in the case of *A. contortum*. Bray & Gibson [9] emphasised the presence of strongly developed ventral musculature in hindbody, a trait exclusive to this genus, suggesting it may have a prehensile function. In fact, *A. contortum* use their suckers and prehensile hindbody to hold onto the host and onto other parasites, thus forming large clusters of parasites. Furthermore, *A. contortum* is not totally exposed to the water in the external microhabitats because the parasites live within pits among host tissue proliferations. It seems that the main adaptation of this species to microhabitats outside the digestive tract involves its capacity to cause intense chronic hyperplasic inflammatory responses, altering its immediate environment, and creating a large proliferation of tissue to cover and protect the parasites from the external environment. Tegumental papillae could be involved in this adaptation to the external environment. The outer dense homogenous layer observed in histological sections over the typical digenean syncitial tegument [45] appears to be a secretion, especially thicker over the papillae. This secretion could be related to the abnormally strong host reaction required to create tissue swellings. Specific stains and TEM sections would be necessary to confirm such glandular activity on the tegument and papillae. Interestingly, these tegumental papillae were not observed in immature parasites living in the digestive tract, where no inflammatory response was detected. These papillae would probably appear in adults when they need to cause strong inflammatory reactions in order to be covered by the host tissues.

## Conclusions

In summary, our study indicates that *A. contortum*, a parasite with the typical trematode morphology, appears to require particular biological adaptations in order to live in the hostile external microhabitats on the host. For this reason, the inflammatory responses caused by these parasites need to seriously alter the gill tissue organization. In high infections with large numbers of *A. contortum*, a wide gill area is damaged thus preventing normal gas exchange, which can even compromise the rearing of the fish in healthy conditions.

### Ethical considerations

The authors declare that no ethical statement or formal approval is required for research on ocean sunfish under the Spanish legislation.
